# Characterization of a Novel Starch Isolated from the Rhizome of Colombian Turmeric (*Curcuma longa* L.) Cultivars

**DOI:** 10.3390/foods13010007

**Published:** 2023-12-19

**Authors:** Shaydier Argel-Pérez, Piedad Gañán-Rojo, Diego Cuartas-Marulanda, Catalina Gómez-Hoyos, Jorge Velázquez-Cock, Lina Vélez-Acosta, Robin Zuluaga, Angélica Serpa-Guerra

**Affiliations:** 1 Programa de Ingeniería en Nanotecnología, Universidad Pontificia Bolivariana, Circular 1, 70-01, Medellin 050031, Colombia; shaydier.argel@upb.edu.co (S.A.-P.); diegocuartasm@outlook.com (D.C.-M.); catalina.gomezh@upb.edu.co (C.G.-H.); jorgeandres.velasquez@upb.edu.co (J.V.-C.); 2 Facultad de Ingeniería Química, Universidad Pontificia Bolivariana, Circular 1, 70-01, Medellin 050031, Colombia; 3 Facultad de Ingeniería Agroindustrial, Universidad Pontificia Bolivariana, Circular 1, 70-01, Medellin 050031, Colombia; lina.velez@upb.edu.co (L.V.-A.); robin.zuluaga@upb.edu.co (R.Z.); angelicamaria.serpa@upb.edu.co (A.S.-G.)

**Keywords:** turmeric, *Curcuma longa* L., starch, *Zingiberaceae*, isolation, characterization, rhizome

## Abstract

Turmeric (*Curcuma longa* L.) plants are native to Southeast Asia and are part of the *Zingiberaceae* family. Global consumption and production of this plant are expanding. In countries such as Colombia, turmeric is a promising cultivar. Curcuminoids derived from its rhizomes are used in food, pharmaceuticals, and natural cosmetics. Curcuminoids constitute approximately 3 wt% of the rhizome. Many residues rich in cellulose and starch can thus be recovered. This study characterizes a novel starch isolated from Colombian turmeric cultivars. The morphological parameters of the starch were determined using microscopic techniques such as scanning electron microscopy (SEM). Proximate analysis and infrared spectroscopy (ATR-FTIR) were used to analyze the chemical composition, while physical analyses included thermal characterization, swelling power testing, solubility, water retention capacity, and colorimetry evaluation. The new starch granules were ellipsoidal in shape and ranged in diameter from 19.91 to 38.09. A trace amount of remaining curcumin was identified through chemical and physical characterization. The swelling power was 3.52 ± 0.30, and its water retention capacity was 3.44 ± 0.30. Based on these findings, turmeric can be useful in both food and non-food applications. Because starch was extracted from other *Zingiberaceae* plants, this study also includes a brief review of the related literature.

## 1. Introduction

Starch is one of the essential components in the human diet, and depending on the region of the world, it represents between 35% to 80% of the daily caloric intake [1]. The global production and use of starch have increased in recent decades. The European Union Member States, for example, increased starch production from 8.7 million tons in 2004 to 10.5 million tons in 2022 [2], while the global market is expected to reach 199.8 million metric tons in 2030 [3].

Beyond the important use of starch for traditional foods [4,5,6], new food and non-food applications of starch are increasing due to their advantages, which include complete biodegradability, widely isolated sources, and ease of modification to vary their physical and chemical properties [7,8]. Recent research topics include flavor and oil encapsulation [8], starch modified with ions for fortified food, and low-cost bio-sorbents for waste waters cleaning [9]. Additionally, there are multiple developments in regards to biodegradable starchy films reinforced with natural fibers or nanoreinforcements that include different forms of cellulose [10,11]. Some of these materials are associated with edible films [12,13], although a predominant focus is their application for functional packaging [14]

The increasing number of applications prompts the constant search for new starch sources, particularly because starch can be isolated from a wide variety of plants, including traditional sources, such as wheat and corn [15], tubers like potatoes [16], and roots like cassava [7]. However, other, less used vegetable sources can be used for its extraction, i.e., sorghum, barley, rice, or the rhizome of plants such as the *Zingiberaceae* [17].

The *Zingiberaceae* family comprises 52 genera and 1300 species of aromatic flowering perennial plants [18]. Ginger (*Zingiber officinale* Roscoe), turmeric (*Curcuma longa* L.), and Javanese ginger (*Curcuma zanthorrhiza* Roxb.) are the most well-known members of this family [18]. These plants are distinguished by their tuberous rhizomes, which contain relevant compounds such as gingerols and curcuminoids, and these compounds are widely used in the food and natural cosmetics industries as aromatic spices, color additives, and flavoring agents [18,19]. These components have been extensively researched due to their antioxidant activity, which has shown promising results in the treatment or control of diabetes and several forms of cancer [18]. As a result, their worldwide consumption is rising. For example, the global commerce in turmeric in 2021 was USD 358 million, with exports increasing by 0.73% over the previous year [20]. India is the world’s largest exporter of turmeric, followed by Myanmar [21].

These demands require the development of new cultivars in other parts of the world, such as the Caribbean and Latin America, where suitable growing conditions exist [22]. In Colombia, over the last ten years, the turmeric cultivar has been promoted by government programs as an alternative to replacing illegal crops, particularly in post-conflict areas [23]. For example, in 2021, 775.12 tons of turmeric were generated on 50.2 hectares of cultivated land in this country [24].

Over the last two decades, there has been an increasing interest in exploiting a higher proportion of the rest of the rhizome, especially considering that starch constitutes 25–70 wt% of the dried rhizome [25,26,27]. This suggests that there is an important opportunity to research starch isolated from the rhizomes of *Zingiberaceae* plants as an additive for use in both food and non-food industries, which could help meet the previously mentioned rising demand.

Considering these options, this study describes the morphological characteristics and the chemical and physical properties of a novel starch isolated from a Colombian *Curcuma longa* L. cultivar. These evaluations include the use of techniques such as electron microscopy, infrared spectroscopy, X-ray diffraction, and thermogravimetric analysis. Additionally, functional characteristics such as swelling power, solubility, water retention capacity, and colorimetry are also considered.

Additionally, in view of the growing interest in these novel and unusual starch resources, and to support future research in this area, this work includes a brief overview of published research on starch isolation from *Zingiberaceae* plants, addressing the questions regarding which countries are leading in this field, which species are being extensively studied, and which methods are being used for its isolation.

## 2. Materials and Methods

### 2.1. Brief Overview

**Figure 1:**
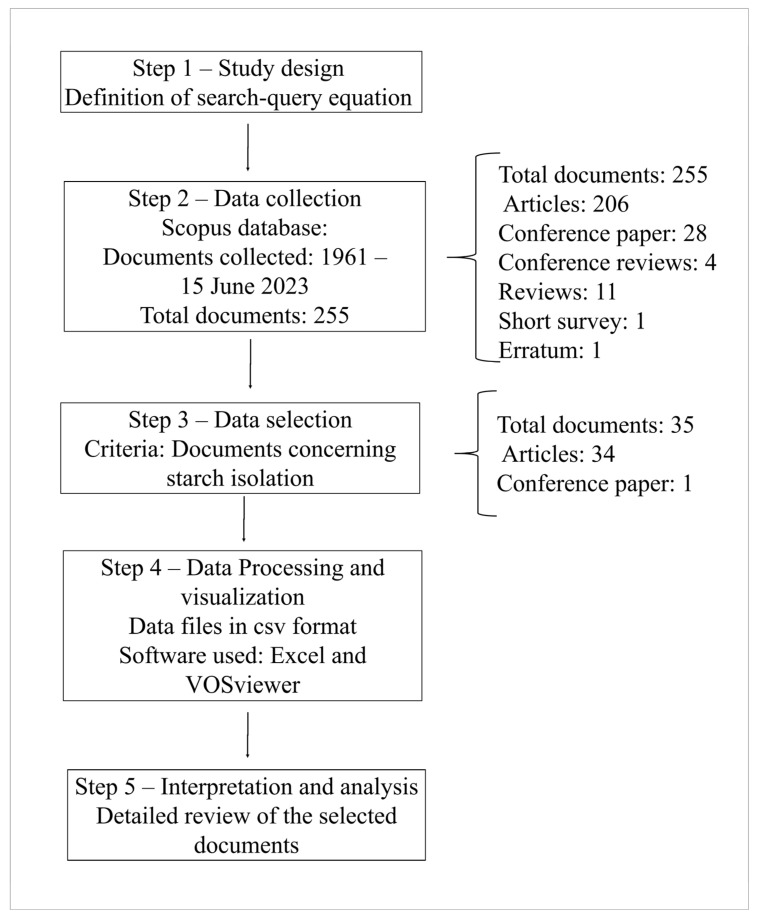
Procedures for performing a bibliometric analysis and a systematic literature review.

This brief overview was carried out to identify the most important research on starch isolation from *Zingiberaceae* species in order to evaulate the starch isolation process and compare the properties of the novel starch isolated. Figure 1 depicts the sequence of the steps. This approach was developed using our prior expertise [28].

As indicated in Figure 1, Step 1 corresponds to the definition of the search-query equation; for this aspect, various prior tests were performed, and the maximum number of documents connected to starch and *Zingiberaceae* species corresponds to “Starch AND (turmeric OR curcuma OR *Zingiberaceae*)”. 

The Scopus database was used to collect the papers in Step 2, as it contained an important number of specialized publications on engineering, chemistry, and food publishing research concerning starch. The search-query equation was used to find the document in the TITLE-ABS-KEY section of the database. Figure 1 shows that 255 documents were found, and 80.8% of the documents correspond to articles.

In Step 3, an exhaustive manual examination of the data enabled the selection of papers containing information about the starch obtained from *Zingiberaceae* species. As seen in Figure 1, 35 documents were found. The remaining documents identified by the search-query equation cover themes such as plant characterization, medical and nutritional applications, biocomposites, and edible film elaboration. In Step 4, each of 35 documents was reviewed to ensure that they included appropriate information about starch isolation.

### 2.2. Isolation of Colombian Turmeric (Curcuma longa L.) Starch

Fresh turmeric rhizomes (*Curcuma longa* L.) were provided by a local farmer in Uramita, Antioquia, Colombia. Colombian varieties are created by combining generic materials imported from other countries. These rhizomes were harvested when they were at their optimum size, which was between 290- and 310 days following planting. To eliminate any remaining dirt and soil, the rhizomes were thoroughly cleaned and disinfected by soaking them for 15 min in a quaternary ammonium solution and then thoroughly rinsing them. The clean rhizomes were peeled and stored at 7 °C until use.

**Figure 2:**
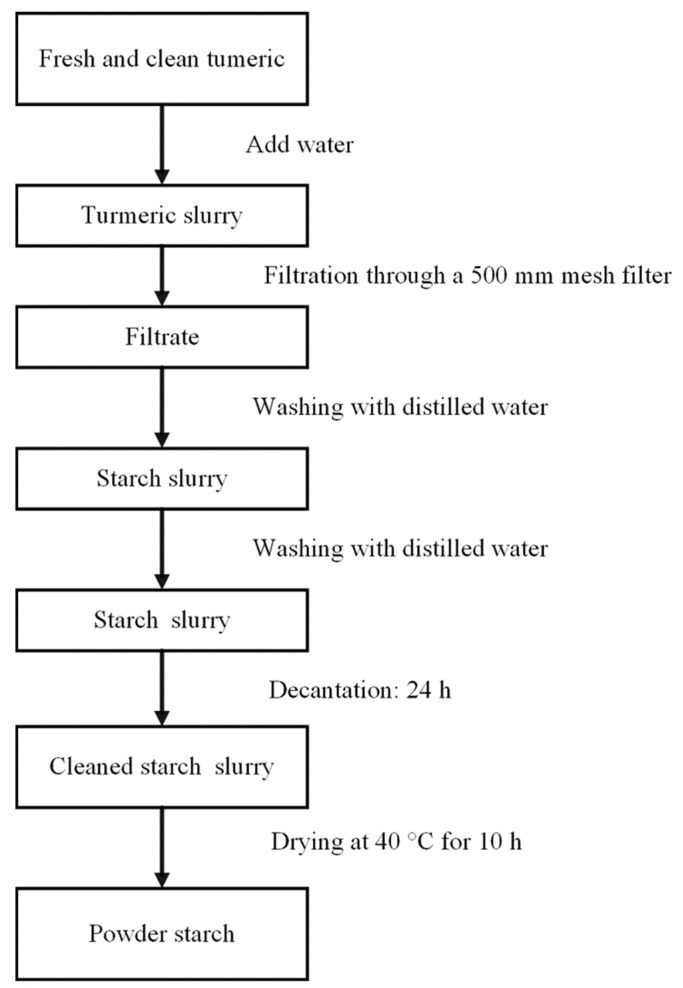
Flow chart of turmeric starch isolation.

Figure 2 displays a scheme of the process used for starch isolation. With minor modifications, this method is based the technique described by Bello-Pérez et al. (1999) [29]. And in general, it is quite similar to other processes that have been published and discussed for the isolation of starch from *Zingiberaceae* plants, as summarized in Table 1. 

The frozen rhizomes were lightly sliced into disks and processed for 3 min at 7000 rpm in a Waring blender, model BL-767, with water in a 1:4 ratio. The turmeric slurry was filtered and rinsed many times with a 500 mm sieve, followed by decantation of the liquid phase for 24 h at room temperature, cleaning, then decanting for another 12 h. Finally, the sample was dried for 10 h in a hot air oven at 40 °C to obtain the starch powder.

### 2.3. Characterization of Colombian Turmeric (Curcuma longa L.) Starch

#### 2.3.1. Morphological Analysis

The microstructure of the extracted starch was analyzed using scanning electron microscopy (SEM). For the analysis, roughly 5 mg of material was deposited on a double-sided adhesive carbon tape and subsequently sputtered with gold (Denton Vacuum Desk V TSC). The samples were analyzed with a Jeol JSM-6000 Plus SEM microscope at a 15 kV acceleration voltage.

A light Nikon Eclipse Ci Microscope equipped with a Nikon DSFI3 camera was used to supplement the morphological study. Some samples were dyed in 20 L of Lugol solution for the contrast examination. For picture analysis, FIJI software 2.3.1—ImajeJ 1.54d was employed [30]. The granule sizes were measured using pictures from both micrographs. A minimum of 50 measurements were carried out to determine the size of the granules.

#### 2.3.2. Chemical Analysis

Proximate analysis was performed according to the AOAC [31], and infrared spectroscopy analysis was performed using an FTIR (Thermo Scientific iS50) coupled with an ATR, with a diamond crystal mounted on tungsten carbide. This technique was used to determine the chemical structure of an analytical grade curcumin standard and isolated turmeric starch. The sample spectra were collected at 64 scans using Omnic 9, version 9.5.9 software at a resolution of 4 cm^−1^. The obtained spectra were processed using the following procedure to eliminate the effects of the crystal and for comparison with the transmission spectra from the equipment’s database:

- Remove the crystal effect by employing the ATR correction advanced algorithm. This correction eliminates the distortion of relative band intensity caused by the dependence of dp on wavelength, the shift of the bands to a lower wavenumber induced by refractive index dispersion, and the deviation from Beer’s Law, provoked by non-polarization effects [32].
- Nine-point smoothing.
- Automatic baseline correction.

To execute the curve fitting in Omnic software, the spectrum was not normalized, and all mathematical treatments were performed in absorbance mode. The goal of spectral curve fitting is to generate individual peaks from a spectrum which, when added together, match the original data. Convergence is the process by which this occurs. Curve fitting involves three steps: selecting initial profiles (line shapes and baseline handling), selecting initial parameters (width, height, and location), and optimization [33].

In the case of Colombian turmeric starch, the initial curve profile selected is Gaussian, which is recommended for solid samples, with high sensitivity, and the FWHH (full width at half height) was maintained at the software’s default of 0.964. In addition, the noise level was defined as 10, which is also determined by the software, and no baseline was defined, since it was previously corrected.

#### 2.3.3. X-ray Diffraction Analysis

Using X-ray diffraction, the crystalline structure of starch was examined. Panalytical X’Pert Pro MPD equipment was used, outfitted with 1.5418 Å, which was the wavelength of the CuKα X-ray. An omega/2θ goniometer was utilized. A reflection transmission spinner running at 4 rpm was the platform configuration. For the diffraction angle 2θ, a range of 10° to 40° was utilized, with a scan speed of 55 s and an angle step of 0.0262606°/min.

#### 2.3.4. Thermal Degradation Analysis

The thermal degradation of starch was evaluated using a Metter Toledo TGA/SDTA 85E thermogravimetric analyzer. A total of 10 mg of the sample was placed in a crucible and examined at a heating rate of 10 °C/min and a temperature range of 25–600 °C in a nitrogen atmosphere of 30 mL/min.

#### 2.3.5. Evaluation of Functional Characteristics

The functional characteristics evaluated for the isolated starch in these studies correspond to swelling power (SP), solubility, and water retention capacity (WRC).

These parameters were measured using the method suggested by Anderson et al. (1970) [34]. In this case, 0.458 g of turmeric starch was placed in a centrifuge tube, and 11 mL of distilled water was added while gently stirring. The tube was placed in a 60 °C water bath for 30 min before being centrifuged at 5000 rpm for another half hour. This gel was weighted and corresponded to the variable gel weight. The supernatant was then decanted, placed in a previously weighed crucible, and dried for 10 h at 40 °C. The remaining samples were weighed again, and the results corresponded to a variable known as the insoluble components weight. Equations (1)–(3) were used to calculate swelling power (SP), solubility, and water retention capacity (WRC).

(1) $$SP=\frac{gel\;weight\;(g)}{\left(turmeric\;starch\;weight\;(g)-insoluble\;components\;weight\;(g)\right)}$$

(2) $$Solubility=\frac{insoluble\;components\;weight\;(g)}{turmeric\;starch\;weight\;(g)}$$

(3) $$WRC=\frac{gel\;weight\;(g)}{turmeric\;starch\;weight\;(g)}$$

#### 2.3.6. Colorimetric Evaluation

An X-Rite SP62 colorimeter was used to produce colorimetric parameters for the purpose of evaluating the residual color in the starch caused by curcuma components, such as curcumin. The parameters are L* = lightness (luminance), or the lightness or darkness of a color; a* = red to green (+a = redder, −a = greener); and b* = yellow to blue (+b = yellower, −b = bluer).

From these values, chroma C* and color hue values were determined using the following equations [35]:

(4) $$C^{*}=\sqrt{\;a^{*2}+b^{*2}}$$

(5) $$h=arctan(\frac{b^{*}}{a^{*}})$$

A minimum of three independent replicates were used to average all numerical results. ANOVA using Excel (Microsoft Office—Windows 11) was performed on the data using Rstudio in order to identify statistically significant (*p* < 0.05) differences.

**Table 1:** Details regarding the basic characteristics and methods of isolation of starches from *Zingiberaceae* plants.

Reference	*Zingiberaceae* Plant Named in the Publication by the Author	Reduction of Size	Isolation Solvent	Yield of the Isolation Process (%)	Starch Granulate Size (μm)	Swelling Power (SP) (g Water/g Starch)	Origin of the Cultivar
Maniglia et al., 2022 [36]	Turmeric dye extraction residue	Milling	Water	30.00 ± 3.00	NR	Around 4.5 (At 65 °C)	Brazil
			Sodium hydroxide	31.00 ± 1.00		Around 6.0 (At 65 °C)	
			Ascorbic acid	24.00 ± 1.00		Around 6.0 (At 65 °C)	
Naidu et al., 2022 [37]	*Curcuma angustifolia* Roxb.	Milling	Water	NR	2.92–6.42	2.4% (At 30 °C)	India
						12.1 (At 80 °C)	
Nakkala et al., 2022 [38]	*Curcuma longa* L.	Milling	Water	NR	NR	6.24 ± 0.31 (At 70 °C)	India
Arini et al., 2021 [39]	Red ginger	Milling	Water	NR	NR	9.09 (At 50 °C)	Indonesia
	Elephant ginger					9.30 (At 50 °C)	
	Emprit ginger					10.31 (At 50 °C)	
	Curcuma					10.53 (At 50 °C)	
Oluba et al., 2021 [40]	Turmeric	Milling	Sodium metabisulphite solution	NR	NR	Swelling capacity 75.1 ± 10.6 (%) (At 50 °C)	Nigeria
Anu et al., 2020 [41]	*Curcuma zanthorrhiza* Roxb.; common name: false turmeric	Milling	Water	10.4 ± 3.7	8.70–39.20	26.16 mL/g (At 90 °C)	India
Tejavathi et al., 2020 [6]	*Curcuma karnatakensis*	Milling	Ammonia solution (0.03 M)	Sample A: 76.4 ± 0.3	1.00–10.00	5.06 ± 0.07 (At 60 °C)	India
				Sample B: 75.0 ± 0.4		5.03 ± 0.04 (At 60 °C)	
Bento et al., 2019 [42]	*Hedychium coronarium* J. Koenig. Common name: white garland lily, butterfly lily, Napoleon, narcissus, Olympia, or white ginger	Milling	Water	22.30 ± 0.30	12.00–38.00	2.22 (At 55 °C)	Brazil
Maniglia and Tapia-Blácido, 2019 [43]	*Curcuma longa* L.	Ball milling and cryogenic milling	Sodium hydroxide	NR	NR	NR	Brazil
			Bleaching using NaClO				
			Bleaching using peroxide hydrogen				
Das and Kumar, 2019 [44]	*Kaempferia galanga* Linn.	Milling	Water	NR	NR	3.62 ± 0.01	India
Silva et al., 2018 [45]	Turmeric–residues after curcuminoids-extract	Milling	Supercritical fluid extraction	NR	NR	NR	Brazil
Franklin et al., 2017 [46]	Commercial *Curcuma angustifolia*	Milling	Commercial sample	NR	6.3–31.7	NR	India
Jamir and Seshagirirao, 2017 [47]	*Curcuma aeruginosa* Roxb.	Milling	Water	NR	6–25		India
	*Curcuma amada* Roxb.				10–30		
	*Curcuma aromatica* Salisb.				5–28		
	*Curcuma caesia* Roxb.				8–30		
	*Kaempferia parviflora* Wall. ex Baker				2–15		
	*Zingiber montanum* (J. Koenig) Link ex A. Diet.				5–20		
Mao et al., 2017 [48]	*Curcuma phaeocaulis* Val	Milling	Water	51.28	NR	Around 2.5	China
	*Curcuma kwangsiensis*			56.88		Around 2.5	
	*Curcuma wenyujin*			54.94		Around 2.5	
	*Curcuma longa* L.			50.56		Around 2.5	
Santana et al., 2017 [49]	Turmeric	Milling	Supercritical fluid extraction using carbon dioxide as the solvent	3.33	NR	NR	Brazil
Van Hung and Vo, 2017 [50]	*Curcuma longa*	Milling	Water	NR	Smaller granules: <20 Larger granlues: 20–50	NR	Vietnam
	*Curcuma caesia*				Smaller granules: <20 Larger granlues: 20–50	NR	
Huang et al., 2015 [51]	*Curcuma longa*	Homogenized with ice-cold sodium metabisulfite solution	Sodium metabisulfite solution	NR	18.6 ± 0.1	Around 2.5	China
Patel et al., 2015 [52]	*Curcuma angustifolia* Roxb. Commonly known as Tikhur	Milling	Water	NR	NR	NR	India
Maniglia et al., 2015 [27]	*Curcuma longa* L.	Milling	Water	NR	10.00–30.00	NR	Brazil
Hansdah et al., 2015 [53]	*Curcuma leucorrhiza*	Milling	Water	NR	30.00–50.00	NR	India
Das et al., 2015 [54]	*Curcuma angustifolia* Roxb., known as Indian Palo	Milling	Water	12.5	Smaller granules: 5.39–7.78	NR	India
Das et al., 2015 [55]	*Curcuma angustifolia* Roxb. known as Indian Palo	Milling	Water	12.5	Smaller granules: 5.39–7.78	2.61 ± 0.01	India
					Larger granules: 25.45–41.56		
Sajitha and Sasikumar, 2015 [56]	*Curcuma amada* Roxb.	Milling	Ammonium oxalate (1 wt%)	48.48 ± 0.31	16–48	4.48 ± 0.04	India
	*Curcuma aromatica* Salisb			45.90 ± 0.10	9–60	3.96 ± 0.05	
	*Curcuma caesia* Roxb.			45.24 ± 0.25	10–39	3.74 ± 0.04	
	*Curcuma xanthorrhiza* Roxb.			46.11 ± 0.18	9–47	4.07 ± 0.01	
Xia et al., 2013 [57]	*Curcuma phaeocaulis* Val.	Milling	Ethanol (95%)	NR	Smaller granulates: 3.00–5.00	NR	
					Larger granulates: 15.00–20.00		China
Rani et al., 2012 [58]	*Curcuma angustifolia* Roxb.	Lab mixer	Ammonium oxalate (1 wt%)	37.64	3.32–32.55	NR	India
		Lab mixer	Ammonia (0.03 M)	38.46			
Kuttigounder et al., 2011 [59]	*Curcuma longa* L.	Lab milling	Water	56	Smaller granules: 3.00–20.00	NR	India
					Larger granules: 20.00–48.00		
Rajeevkumar et al., 2010 [60]	*Curcuma angustifolia*	Milling	Water	27.5	9.86	11.29	India
Ascheri et al., 2010 [61]	*Hedychium coronarium*	Milling	Water	NR	11.80–52.73	NR	Brazil
Policegoudra and Aradhya, 2008 [62]	*Curcuma amada* Roxb.	Milling	Water	NR	Smaller granules: 3.00–20.00	NR	India
					Larger granules: 20.00–48.00		
Ibezim et al., 2008 [63]	*Zingiber officinale*	Milling	Water	NR	NR	NR	Nigeria
Ranjini and Vijayan, 2006 [64]	*Curcuma aeruginosa*	Milling	Water	NR	NR	NR	India
Braga et al., 2006 [26]	*Curcuma longa* L.	Milling	Sodium hydroxide (0.25 wt%) - Supercritical fluid extraction	NR	10.00–33.00	2.11 ± 0.04	Brazil
Moreschi et al., 2006 [65]	*Curcuma longa* L.	Milling	Subcritical fluid extraction with water and CO_2_	NR	10.00–33.00	NR	Brazil
Jyothi et al., 2003 [66]	*Curcuma zedoaria*	Milling	Water	NR	Smaller granules: 3.00–30.00	Swelling volume (mL/g) 14.8 ± 1.2	India
					Larger granules: 35.00–60.00		
	*Curcuma malabarica*				Smaller granules: 9.00–30.00	Swelling volume (mL/g) 22.3 ± 0.5	
					Larger granules: 30.00–45.00		
Leonel et al., 2003 [25]	*Curcuma longa* L.	Milling	Water	NR	20–25	NR	Brazil
	*Curcuma zedoaria*				20–30		

Note: NR stands for “information not reported by authors” throughout the text.

## 3. Results and Discussion

### 3.1. Brief Overview

A review of starches extracted from *Zingiberaceae* plants is provided before examining the results of the novel starch isolated from the Colombian cultivar of turmeric (*Curcuma longa* L.).

Table 1 summarizes the species of the *Zingiberaceae* plant used to isolate starch, along with the extraction method, the granulate size, the swelling power of the isolated product, and the country in which the plant was grown. As can be observed, Curcuma is the most often occurring genus, appearing in 81.5% of all studies. Approximately 29.6% of the study under consideration focused on the species *C. longa* L. *Zingiber*, which is the second most commonly used genus, accounting for 11.0% of all studies.

The studies were conducted in six different countries spread across three continents. The majority of studies (28.5%) were conducted on Brazilian samples, followed by Indian samples (22.9%). It is important to note that 68.29% of the starch samples reported in Table 1 come from the three major turmeric exporting countries of 2021, i.e., India (62.10% of the studies), Vietnam (3.69%), and Indonesia (2.5%). Nigeria’s scenario is remarkable because a number of studies have been published regarding this country, despite Nigeria’s low export participation rate of 0.65% in 2021. However, according to Table 1, 31.43% of the studies used samples from Brazil, despite the fact that this country is only one of the top 50 exporters of turmeric in 2021. This interest in curcuma research could be attributed to the significant use of turmeric in this country [20].

In terms of starch isolation technologies, the need to reduce the size of the plant sources were highlighted in all the research. In all situations, a form of milling technique was adopted which included, for example, lab milling [60], also known as ball milling, and cryogenic milling [44]. Water was utilized as the starch isolation solvent in 65.7% of the studies. However, in certain cases, the authors used other alternatives, including supercritical fluids [49] or a solution of sodium hydroxide in water [26]. One benefit of using water solvent is that exerts less impact on the environment. For this reason, in this study, this solvent was chosen for the isolation of starch from Colombian curcuma cultivars.

### 3.2. Colombian Turmeric (Curcuma longa L.) Starch

In this case, the yield of starch isolation corresponds to 12.2 ± 1.0%. This value is comparable to that reported for *Curcuma zanthorrhiza* Roxb [41] and *Curcuma angustifolia* Roxb [54,55], whereas other starches isolated from turmeric or *Curcuma longa* exhibit higher values [36,48,59]. These variations can be attributed to crop factors and turmeric maturity [67].

**Figure 3:**
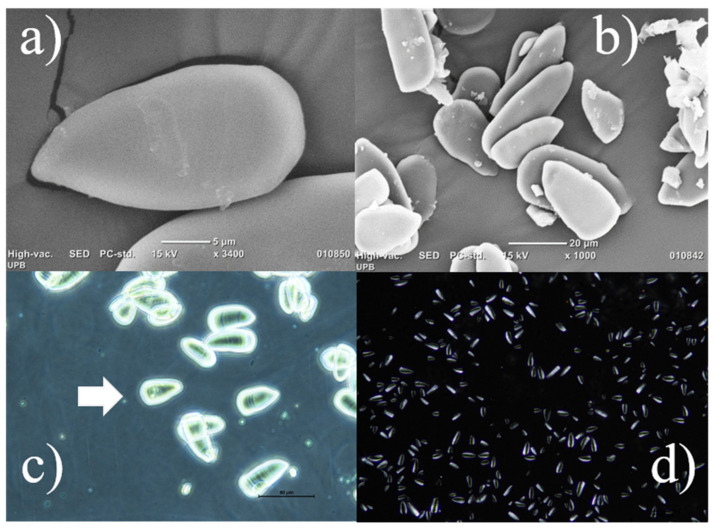
Starch micrographs: SEM 1400X (**a**); SEM 1000X (**b**). Optical microscopy image by phase contrast 40X (**c**) and polarized light (**d**).

As shown in Figure 3a, starch granules isolated from turmeric rhizomes have an ellipsoidal shape with triangular features, are thin, and have a smooth surface, and no cracks were observed due to isolation. As seen in Figure 3b, the diameters vary between 19.91 and 38.09 µm, with a mean value of 29.0 µm. 

The granulate sizes, as indicated in Table 1, are on the same scale as other values reported for starch extracted from *Zingiberaceae* plants, and the granules can be classified as larger granules. These results show that starch granules had a narrower spread size distribution than the other examples listed in Table 1. 

The phase-contrast method and polarized light microscopies were used to examine the starch granules. The malt crosses are not visible in Figure 3c,d, but the striae curves are, especially in the phase contrast images (Figure 3c). These findings suggest that starch has a low crystallinity; a similar finding was reported by Bento et al. (2019) [42] for starch isolated from white garland lily rhizomes.

The hilums that are encircled by growth rings in Figure 3c are another distinctive morphological feature. According to certain writers [67], this shape is connected to rhizome maturity and cultivar environments. In this case, the Colombian plants were grown in a humid zone under monsoon weather conditions, such as the Uramita region, which can explain this starch structure.

According to Figure 3a–d, the starch granulates are separated, do not show a higher presence of other compounds, and exhibit no visible damage. These findings contrast sharply with those of Maniglia et al. [36], who noticed that the presence of non-starchy rhizome components facilitated starch agglomeration. According to these results, the novel isolated starch may be advantageous in the formation of films, with the potential application to biocomposites or edible films [13], as well as a matrix for biomolecule encapsulation [59].

Table 2 shows the results of the proximal analysis of turmeric starch. The moisture content of isolated turmeric starch is higher than that of other *Zingiberaceae* species [47]. The lipid and protein contents, however, are comparable to those of other starches isolated from turmeric samples [25], as well as tapioca, corn, or potato [68]. Again, these discrepancies could, once again, be attributed to cultivar conditions. This issue can be examined further in future studies in this field.

**Figure 4:**
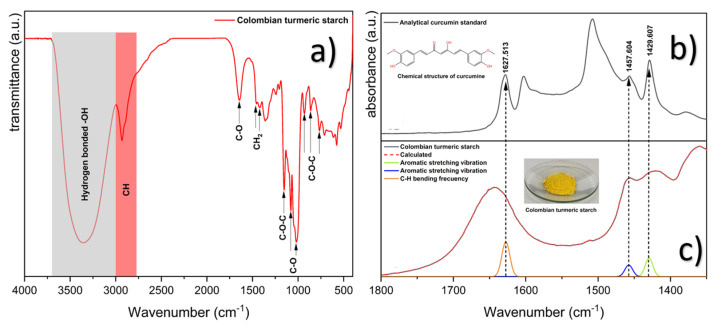
Infrared spectrum of the analytical standard of curcumin and Colombian turmeric. Starch: Colombian turmeric starch (**a**); analytical standard of curcumin (**b**); mathematical adjustment in the 1800–1300 cm^−1^ range to determine the absorbances associated with residual curcumin present in Colombian turmeric starch (**c**).

In Figure 4a, the infrared spectrum of a yellow powdered sample is observed. Upon comparison with the database in the infrared equipment software, it was determined to have a 91.92% similarity to a spectrum of pure starch (HR inorganics library with identification number 1464). This confirms the presence of a starch-rich sample obtained from Colombian turmeric.

Colombian turmeric starch exhibits a broad zone between 3700–3000 cm^−1^ (gray shade in Figure 4a) associated with the stretching vibration of hydrogen bonds [69,70,71]. Additionally, a band in the region between 3000–2800 cm^−1^ (red shade in Figure 4a) can be assigned largely by the contribution from the CH stretching vibration and also partially from the CH_2_ stretching vibration of the CH_2_OH group in each glucose residue [69,71,72], while a vibration at 1644 cm^−1^ is assigned in native starch to the scissoring of two O-H bonds of absorbed water molecules in the non-crystalline structure [69,72]. Bands around 1458, 1423 cm^−1^ are linked to the symmetric deformation of CH_2_ [72]. Furthermore, vibrations at 1153 cm^−1^ are observed, which are associated with the asymmetric stretching of C-O-C [73], along with peaks at 1079 cm^−1^ and 1019 cm^−1^ associated with C-O stretching [73]. Three bands at 932, 872, and 766 cm^−1^ are associated with the vibration of the C-O-C ring in the carbohydrate derived from α-glucopyranose [72,74].

As mentioned earlier, the sample exhibits a yellow color that may be associated with the presence of residual curcumin. To confirm this, Figure 4b,c present the infrared spectrum of an analytical standard of curcumin as a reference to identify the main absorbances associated with this compound, along with the deconvolution in the range between 1800–1300 cm^−1^ of Colombian turmeric starch. The latter is necessary due to the overlap of absorbances that complicates their analysis. Upon examination, three typical curcumin vibrations are identified. Absorbances around 1627 cm^−1^ are associated with the frequency of flexion of the aromatic harmonic [75], and those at 1457 cm^−1^, 1429 cm^−1^ with aromatic vibrations of stretching in the benzene ring [75], confirming the presence of residual curcumin. Furthermore, as noted by other writers like Manigilia et al. [36] and Priyanka [76], the vibration at 1510 cm^−1^ can be connected to the aromatic ring of curcuminoids.

The vibrations at 1152 and 1078 cm^−1^ are related to the stretching vibrations of the C-O bond of the aldehyde group [36], and the vibration at 1019 cm^−1^ is related to the vibration stretching of the C-O bond in C-O-C at the anhydroglucose repeat unit of the starch [77], as well as of polysaccharides [42]. The large vibration at 1019 cm^−1^ is also associated with the amorphous region [42]. This amorphous zone may assist in explaining why it was difficult to see the malt crosses using polarized light microscopy.

Other starch vibrations correspond to 861 and 765 cm^−1^ associated with the C-O-H and C-O-C glycids of α-linked bonds [42]. Other polysaccharides can be associated with vibrations at 931 cm^−1^, 861 cm^−1^, and 533 cm^−1^ [71].

**Figure 5:**
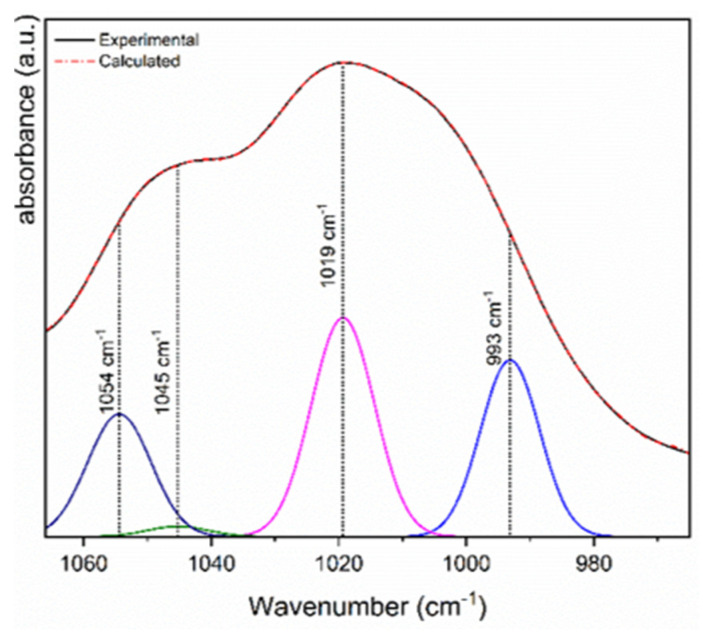
Deconvolution of the region between 1066–960 cm^−1^ for Colombian turmeric starch.

On the other hand, it has been demonstrated by ATR-FTIR that vibrations in the range between 1100 and 970 cm^−1^, attributed to the stretching of C-O and C-C bonds, can be sensitive to changes in the physical state of the starch structure, particularly associated with the crystalline order [71,73]. Figure 5 shows the deconvolution of the region between 1066–960 cm^−1^ for Colombian turmeric starch, where the main absorbances at 1054, 1045, 1019, and 993 cm^−1^ are observed. The ratio of 1054/1019 cm^−1^ was employed to quantify the degree of the short-range ordered structure, obtaining a value of 0.6, a range comparable to the values of type B polymorphism starch [71]. These findings are consistent with those of starch samples that were isolated from various *Zingiberaceae* plants [42,71,78]. 

**Figure 6:**
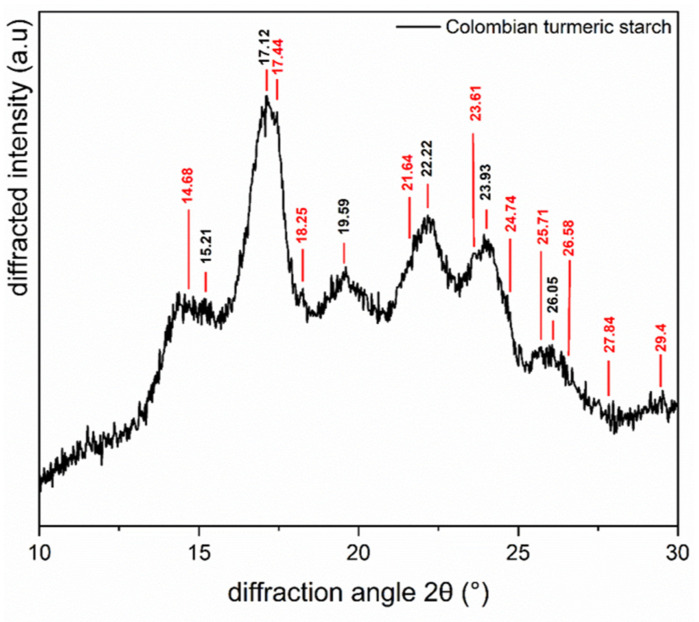
X-ray diffraction of Colombian turmeric starch.

Figure 6 shows the X-ray diffractogram of the isolated Colombian turmeric starch. The main peaks that appear at 2θ are 15.21°, 17.12°, 19.59°, 22.22°, 23.93°, and 26.05° are associated with starch B-type polymorphism, which consists of double helices grouped in a hexagon with an open structure and a hydrated core [71]. 

The intensity of the peak at 19.59° can be attributed to interactions with the amylose and lipids that remain in the isolated starch, as well as with the other polysaccharides [36]; nevertheless, the intensity of the peaks highlighted in red in Figure 6 at 14.68°, 17.44°, 18.25°, 21.64°, 23.61°, 24.74°, 25.71°, 26.58°, 27.84°, and 29.4° can also be attributed to the presence of curcuminoids [36,75,79,80], which is in accordance with the analyses found through ATR-FTIR. Other polysaccharide-related peaks include those at 17.12° and 22.22° [36,45,81]. The findings are consistent with those previously noted in the proximal analysis.

**Figure 7:**
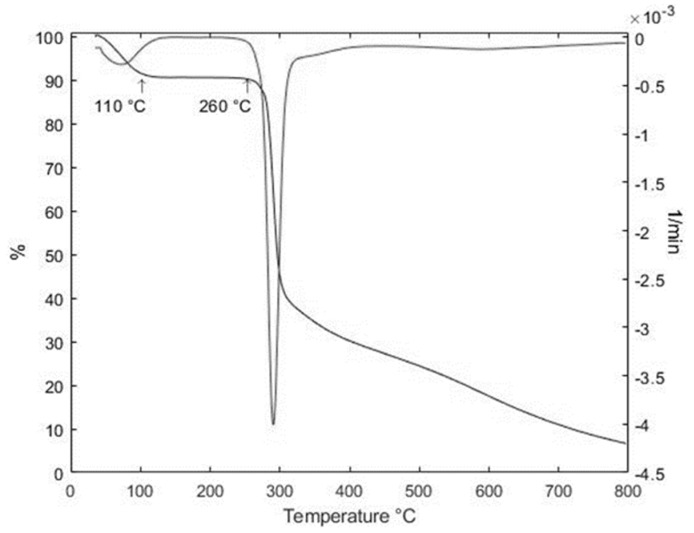
Thermal analysis of the Colombian turmeric starch.

Figure 7 shows the thermal analysis of the separated starch. There were three substantial thermal events identified. The first event takes place between 25 and 212 °C and is related to moisture content, while the second occurs between 260 and 400 °C and is related to starch macromolecule depolymerization. This decomposition is typical of homopolysaccharides such as starch [42]. Thermal degradation occurs at 290 °C. The third event begins at 400 °C, and the residues are less than 10% by 800 °C. This decomposition region is related to organic material oxidation [55]. These results are comparable to those reported by Das et al. [55] in their investigation on the starch extracted from *Curcuma angustifolia* Roxb. This result confirms that the non-starchy components mentioned above are present in both the FTIR evaluation and the proximate analysis.

Swelling power and solubility are directly correlated with hydrophilicity and water-holding capacity [44]. Because each application requires different characteristics and paste performance, evaluating these starch properties aids in understanding the quality of the isolated starches. Therefore, the results of the swelling power, solubility, and water retention capacity determinations of turmeric starch are provided in Table 2.

As indicated in Table 2, the swelling power values are higher than those reported in previous investigations on starch isolated from *Curcuma longa* plants [26,48,51]. However, the swelling power is lower than the value obtained for curcuma in the studies by Arini et al. [39] and Nakkala et al. [38] studies. These differences could be attributed to the content of amylopectin, the major component of the crystalline sections of the granules that conditions starch expansion, as well as the presence of various components such as lipids [36].

Table 2 shows that the water solubility of *Curcuma longa* is lower than that reported by Nakkala et al. [38]. This variance can be attributed to an amylose–lipid complex, which resulted in amylose leaching and reduced starch solubility [36]. 

Given that the swelling value influences starch behavior during cooking and can be used to predict the cooking and eating quality of starch foods [82], the value reported by Colombian turmeric isolated starch suggests that starch can be useful in food formulations that require low cooking losses. This means that it can be used in the formulation of starchy noodles and comparable products, as well as in bakery products which require strict final volume control [83].

As shown in Table 2, Colombian turmeric starch has a lower water retention capacity or water binding capacity than other starches, such as potato (28.85 g/g), canna (6.63 g/g), or corn (9.59 g/g) [84]. This result suggests that this starch is unsuitable for food products that experience syneresis during storage [84], although it may be suited for preserving the freshness of bread products or sausages [41].

Table 2 shows the color evaluation, with the value of L* corresponding to 67.66, indicating that isolated starch can be perceived as a clear sample with high luminosity. The values of a* and b* suggest a hue between red and yellow, with a larger bias toward yellow, and the value of C* indicates an important level of color intensity. Because the angle of the hue was 1.29°, it is regarded to have a reddish hue. These results support the presence of the curcuminoids mentioned previously in the ATR-FTIR results. According to Naidu et al. [37], the white appearance of extracted starch from curcuma sources can be modified through the application of mechanical procedures, such as centrifugation.

In a broader sense, the starch isolated from the rhizome of Colombian turmeric (*Curcuma longa* L.) contains non-starch components, particularly curcuminoids, and exhibits the characteristic B polymorphism starch found in other *Zingiberaceae* plants. The variations in morphological and physical properties compared to other starches isolated from Curcuma longa may be due to cultivation factors and the maturity of turmeric. This effect could be investigated further in future research.

## 4. Conclusions

The morphological, chemical, and physical characteristics of a new starch isolated from Colombian turmeric were evaluated in this work. A brief review of the starch obtained from *Zigiberaceae* plants was conducted in order to compare the results obtained and to identify the most commonly used technologies for isolating starch from these plants. The chemical and physical characteristics of the novel isolated starch are comparable to those of previously isolated starches from several species of turmeric, such as elliptical granule shapes and a distinctive B-type polymorphism, as confirmed by ATR-FTIR spectroscopy and X-ray diffraction. Minor granule crystallinity differences, such as the absence of visible malt crosses, can be attributed to cultivar conditions, which in this case, refer to tropical and humid conditions. Curcuminoids, on the other hand, are still present in low concentrations in the starch samples, as indicated by the chemical and physical evaluations, but they can be removed, depending on the application. Other non-starch components which could exist at lower concentrations than those of curcuminoids could require more sensitive characterization techniques than those used in this study. Our results suggest that starch obtained from Colombian turmeric can be used to develop edible films or starchy foods that require the preservation of freshness, such as bread.

## Figures and Tables

**Table 2:** Chemical composition and physical characteristics of turmeric starch.

Variable	Units	Result
Chemical composition		
Carbohydrates	g/100 g	83.69
Ashes	g/100 g	0.82
Fat	g/100 g	0.05
Protein	g/100 g	0.44
Moisture	g/100 g	15.00
Physical characteristics		
Swelling power (SP)	g water/g starch	3.52 ± 0.30
Solubility	wt%	2.41 ± 0.10
Water retention capacity (WRC)	g gel/g starch	3.44 ± 0.30
Color evaluation		
L*		67.66 ± 0.49
a*		21.25 ± 0.19
b* (WRC)		74.48 ± 0.47
C*		77.45 ± 0.44

## Data Availability

The data presented in this study are available on request from the corresponding author.

## References

[1] Burrell M.M. (2003). Starch: The need for improved quality or quantity—An overview. J. Exp. Bot..

[2] Starch Europe (2023). The European Starch Industry. A Crucial Link between Farm and Fork. https://starch.eu/the-european-starch-industry/.

[3] Starch: Global Strategic Business Report Report. January 2023 Region: Global Industry Analysts, Inc.. https://www.researchandmarkets.com/reports/2832332/starch-global-strategic-business-report?utm_source=GNOM&utm_medium=PressRelease&utm_code=czgx4c&utm_campaign=1825573+-+Starch+Global+Market+to+Reach+199.8+Million+Metric+Tons+by+2030%3a+Use+of+Starch+as+a+Fat+Replacer+Drives+Growth&utm_exec=jamu273prd-global?w=12.

[4] Omoregie Egharevba H., Emeje M. (2019). Chemical properties of starch and its application in the food industry. Chemical Properties of Starch.

[5] Zhu J., Bai Y., Gilbert R.G. (2023). Effects of the molecular structure of starch in foods on human health. Foods.

[6] Tejavathi D.H., Sujatha B.S., Karigar C.S. (2020). Physicochemical properties of starch obtained from *Curcuma karnatakensis*—A new botanical source for high amylose content. Heliyon.

[7] Falua K.J., Pokharel A., Babaei-Ghazvini A., Ai Y., Acharya B. (2022). Valorization of starch to biobased materials: A review. Polymers.

[8] Wang X., Yuan Y., Yue T. (2015). The application of starch-based ingredients in flavor encapsulation. Starch.

[9] Szymońska J., Molenda M., Wieczorek J. (2015). Study of quantitative interactions of potato and corn starch granules with ions in diluted solutions of heavy metal salts. Carbohydr. Polym..

[10] Dufresne A., Vignon M.R. (1998). Improvement of starch film performances using cellulose microfibrils. Macromolecules.

[11] Montoya U., Zuluaga R., Castro C., Vélez L., Gañán P. (2019). Starch and starch/bacterial nanocellulose films as alternatives for the management of minimally processed mangoes. Starch.

[12] Maniglia B.C., Domingos J.R., de Paula R.L., Tapia-Blácido D.R. (2014). Development of bioactive edible film from turmeric dye solvent extraction residue. LWT.

[13] Istiqomah A., Rahmi Utami M., Firdaus M., Suryanti V., Kusumaningsih T. (2022). Antibacterial chitosan-*Dioscorea alata* starch film enriched with essential oils optimally prepared by following response surface methodology. Food Biosci..

[14] Huang J., Wu W., Niu B., Fang X., Chen H., Wang Y., Gao H. (2023). Characterization of *Zizania latifolia* polysaccharide-corn starch composite films and their application in the postharvest preservation of strawberries. LWT.

[15] Ottum E., Baker S.E., Bredeweg E.L. (2021). Production of Biofuels from Biomass by Fungi. Encyclopedia of Mycology.

[16] Lee B.-H., Lee Y.-T. (2017). Physicochemical and structural properties of different colored sweet potato starches. Starch.

[17] Horstmann S., Lynch K., Arendt E. (2017). Starch characteristics linked to gluten-free products. Foods.

[18] Alolga R.N., Wang F., Zhang X., Li J., Tran L.-S.P., Yin X. (2022). Bioactive compounds from the *Zingiberaceae* family with known antioxidant activities for possible therapeutic uses. Antioxidants.

[19] Serpa Guerra A., Gómez Hoyos C., Velásquez-Cock J.A., Vélez Acosta L., Gañán Rojo P., Velásquez Giraldo A.M., Zuluaga Gallego R. (2020). The nanotech potential of turmeric (*Curcuma longa* L.) in food technology: A review. Crit. Rev. Food Sci..

[20] OEC Turmeric (Curcuma). https://oec.world/en/profile/hs/turmeric-curcuma.

[21] (2023). Consumer Goods & FMCG Food & Nutrition. https://www.statista.com/statistics/798287/main-turmeric-export-countries-worldwide/#:~:text=Leading%20global%20exporting%20countries%20of%20turmeric%202022&text=India%20is%20y%20far%20the,exporter%20of%20turmeric%2C%20the%20Myanmar.

[22] Food and Agriculture Organization of the United Nations (FAO) (2004). Turmeric Post-Harvest Operations. http://www.fao.org/3/aau994e.pdf.

[23] United Nations Office on Drugs and Crime (2013). Informe Ejecutivo: Encuentro Nacional de Desarrollo Alternativo. https://www.unodc.org/documents/colombia/2014/Agosto/EncuentroDA/ENDA_2013_ESPANOL.pdf.

[24] Agronet Reporte: Área, Producción y Rendimiento Nacional por Cultivo. https://agronet.gov.co/estadistica/Paginas/home.aspx?cod=1.

[25] Leonel M., Sarmento S.B.S., Cereda M.P. (2003). New starches for the food industry: *Curcuma longa* and *Curcuma zedoaria*. Carbohydr. Polym..

[26] Braga M.E.M., Moreschi S.R.M., Meireles M.A.A. (2006). Effects of supercritical fluid extraction on *Curcuma longa* L. and *Zingiber officinale* R. starches. Carbohydr. Polym..

[27] Maniglia B.C., De Paula R.L., Domingos J.R., Tapia-Blácido D.R. (2015). Turmeric dye extraction residue for use in bioactive film production: Optimization of turmeric film plasticized with glycerol. LWT.

[28] Gañán P., Barajas J., Zuluaga R., Castro C., Marín D., Tercjak A., Builes D.H. (2023). The evolution and future trends of unsaturated polyester biocomposites: A bibliometric analysis. Polymers.

[29] Bello-Pérez L., Agama-Acevedo E., Sánchez-Hernández L., Paredes-López O. (1999). Isolation and partial characterization of banana starches. J. Agric. Food Chem..

[30] Rueden C.T., Schindelin J., Hiner M.C., DeZonia B.E., Walter A.E., Arena E.T., Eliceiri K.W. (2017). ImageJ2: ImageJ for the next generation of scientific image data. BMC Bioinform..

[31] Arzapalo Quinto D., Huamán Cóndor K., Quispe Solano M., Espinoza Silva C. (2015). Extracción y caracterización del almidón de tres variedades de quinua (*Chenopodium quinoa* Willd) Negra collana, Pasankalla roja y Blanca junín. Rev. Soc. Química Perú.

[32] Nunn S., Nishikida K. (2008). Advanced ATR Correction Algorithm.

[33] Bradley M. (2007). Curve Fitting in Raman and IR Spectroscopy: Basic Theory of Line Shapes and Applications.

[34] Anderson R.A., Conway H.F., Peplinski A.J. (1970). Gelatinization of corn grits by roll and extrusion cooking. Starch.

[35] Sappi Fine Paper North America (2013). Defining and Communicating Color: The CIELAB System. https://cdn-s3.sappi.com/s3fs-public/sappietc/Defining%20and%20Communicating%20Color.pdf.

[36] Maniglia B.C., Garcia Silveira T.M., Tapia-Blácido D.R. (2022). Starch isolation from turmeric dye extraction residue and its application in active film production. Int. J. Biol. Macromol..

[37] Naidu M.M., Shilpa H.N., Maheshwari S.U., Sruthi P. (2022). Studies on physico-chemical structural and functional properties of tikhur (*Curcuma angustifolia* Roxb) starch. Trends Carbohydr. Res..

[38] Nakkala K., Godiyal S., Ettaboina S.K., Laddha K.S. (2022). Chemical modifications of turmeric starch by oxidation, phosphorylation, and succinylation. Starch.

[39] Arini T., Yusraini E., Lubis Z. (2021). Functional characteristics of starch from red ginger, elephant ginger, emprit ginger and curcuma. IOP Conf. Ser. Earth Environ. Sci..

[40] Oluba O.M., Osayame E., Shoyombo A.O. (2021). Production and characterization of keratin-starch bio-composite film from chicken feather waste and turmeric starch. Biocatal. Agric. Biotechnol..

[41] Anu S., Dan M., Suja S.R. (2020). Wild relative of turmeric, *Curcuma zanthorrhiza* Roxb.—A source of edible starch. Indian J. Tradit. Knowl..

[42] Bento J.A.C., Ferreira K.C., de Oliveira A.L.M., Lião L.M., Caliari M., Júnior M.S.S. (2019). Extraction, characterization and technological properties of white garland-lily starch. Int. J. Biol. Macromol..

[43] Maniglia B.C., Tapia-Blácido D.R. (2019). Structural modification of fiber and starch in turmeric residue by chemical and mechanical treatment for production of biodegradable films. Int. J. Biol. Macromol..

[44] Das D., Kumar K.J. (2019). Enhancing resilient property of *Kaempferia galanga* rhizome starch by succinylation. Int. J. Biol. Macromol..

[45] Silva E.K., Martelli-Tosi M., Vardanega R., Nogueira G.C., Zabot G.L., Meireles M.A.A. (2018). Technological characterization of biomass obtained from the turmeric and annatto processing by using green technologies. J. Clean. Prod..

[46] Franklin M.E.E., Pushpadass H.A., Kumar B., Kulkarni S., Muthurayappa M., Kandasamy R., Venkatachalam P., Vellingiri P. (2017). Physicochemical, thermal, pasting, and microstructural characterization of commercial *Curcuma angustifolia* starch. Food Hydrocoll..

[47] Jamir K., Seshagirirao K. (2017). Isolation, characterization and comparative study of starches from selected *Zingiberaceae* species, a non-conventional source. Food Hydrocoll..

[48] Mao X., Huang H., Li X., Wang T., Chen X., Gao W. (2017). Physicochemical characterization, digestibility and anticonstipation activity of some high-resistant untraditional starches from *Zingiberaceae* plants. Int. J. Food Sci. Technol..

[49] Santana Á.L., Zabot G.L., Osorio-Tobón J.F., Johner J.C.F., Coelho A.S., Schmiele M., Steel C.J., Meireles M.A.A. (2017). Starch recovery from turmeric wastes using supercritical technology. J. Food Eng..

[50] Van Hung P., Vo T.N.D. (2017). Structure, physicochemical characteristics, and functional properties of starches isolated from yellow (*Curcuma longa*) and black (*Curcuma caesia*) turmeric rhizomes. Starch.

[51] Huang J., Zhao L., Man J., Wang J., Zhou W., Huai H., Wei C. (2015). Comparison of physicochemical properties of B-type nontraditional starches from different sources. Int. J. Biol. Macromol..

[52] Patel S., Tiwari S., Pisalkar P.S., Mishra N.K., Naik R.K., Khokhar D. (2015). Indigenous processing of Tikhur (*Curcuma angustifolia* Roxb.) for the extraction of starch in Baster, Chhattisgarh. Indian J. Nat. Prod. Resour..

[53] Hansdah R., Prabhakar P.K., Srivastav P.P., Mishra H.N. (2015). Physico-chemical characterization of lesser known Palo (*Curcuma leucorrhiza*) starch. Int. Food Res. J..

[54] Das D., Jha S., Kumar K.J. (2015). Effect of carboxymethylation on physicochemical and release characteristics of Indian Palo starch. Int. J. Biol. Macromol..

[55] Das D., Jha S., Kumar K.J. (2015). Isolation and release characteristics of starch from the rhizome of Indian Palo. Int. J. Biol. Macromol..

[56] Sajitha P.K., Sasikumar B. (2015). Qualitative and quantitative variation in starch from four species of Curcuma. Cytologia.

[57] Xia Y., Gao W., Wang H., Jiang Q., Li X., Huang L., Xiao P. (2013). Characterization of tradition Chinese medicine (TCM) starch for potential cosmetics industry application. Starch.

[58] Rani A., Chawhaan P.H. (2012). Extraction and scanning electron microscopic studies of *Curcuma angustifolia* Roxb. Starch.

[59] Kuttigounder D., Rao Lingamallu J., Bhattacharya S. (2011). Turmeric powder and starch: Selected physical, physicochemical, and microstructural properties. J. Food Sci..

[60] Rajeevkumar P., Rajeev R.E.K.H.A., Anilkumar N. (2010). Studies on *Curcuma angustifolia* starch as a pharmaceutical excipient. Int. J. PharmTech Res..

[61] Ascheri D.P.R., de Souza Moura W., Ascheri J.L.R., de Carvalho C.W.P. (2010). Caracterização física e físico-química de rizomas e amido do lírio-do-brejo (*Hedychium coronarium*). Pesqui. Agropecu..

[62] Policegoudra R.S., Aradhya S.M. (2008). Structure and biochemical properties of starch from an unconventional source—Mango ginger (*Curcuma amada* Roxb.) rhizome. Food Hydrocoll..

[63] Ibezim E.C., Ofoefule S.I., Omeje E.O., Onyishi V.I., Odoh U.E. (2008). The role of ginger starch as a binder in acetaminophen tablets. J. Sci. Res. Essays.

[64] Ranjini C.E., Vijayan K.K. (2006). A high amylose starch isolated from the tubers of *Curcuma aeruginosa*. Indian J. Chem.—B Org. Med..

[65] Moreschi S.R.M., Leal J.C., Braga M.E.M., Meireles M.A.A. (2006). Ginger and turmeric starches hydrolysis using subcritical water+ CO_2_: The effect of the SFE pre-treatment. Braz. J. Chem. Eng..

[66] Jyothi A.N., Moorthy S.N., Vimala B. (2003). Physicochemical and functional properties of starch from two species of Curcuma. Int. J. Food Prop..

[67] Svegmark K., Hermansson A.M. (1993). Microstructure and rheological properties of composites of potato starches granules and amylose: A comparison of observed and predicted structure. Food Struct..

[68] Hernández-Medina M., Torruco-Uco J.G., Chel-Guerrero L., Betancur-Ancona D. (2008). Caracterización fisicoquímica de almidones de tubérculos cultivados en Yucatán, México. Food Sci. Technol..

[69] Liu J., Chen J., Dong N., Ming J., Zhao G. (2012). Determination of degree of substitution of carboxymethyl starch by Fourier transform mid-infrared spectroscopy coupled with partial least squares. Food Chem..

[70] Hong J., Chen R., Zeng X.-A., Han Z. (2016). Effect of pulsed electric fields assisted acetylation on morphological, structural and functional characteristics of potato starch. Food Chem..

[71] Pozo C., Rodríguez-Llamazares S., Bouza R., Barral L., Castaño J., Müller N., Restrepo I. (2018). Study of the structural order of native starch granules using combined FTIR and XRD analysis. J. Polym. Res..

[72] Horitsu K. (1960). Physicochemical Properties: On Infrared Absorption Spectra of Native Starch and Modified Starch using Film. Bull. Agric. Chem. Soc. Jpn..

[73] Van Soest J.J., Tournois H., de Wit D., Vliegenthart J.F. (1995). Short-range structure in (partially) crystalline potato starch determined with attenuated total reflectance Fourier-transform IR spectroscopy. Carbohydr. Res..

[74] Paluch M., Ostrowska J., Tyński P., Sadurski W., Konkol M. (2022). Structural and thermal properties of starch plasticized with glycerol/urea mixture. J. Polym. Environ..

[75] Hettiarachchi S.S., Dunuweera S.P., Dunuweera A.N., Rajapakse R.M.G. (2021). Synthesis of curcumin nanoparticles from raw turmeric rhizome. ACS Omega..

[76] Priyanka S.K. (2018). Influence of operating parameters on supercritical fluid extraction of essential oil from turmeric root. J. Clean. Prod..

[77] Arik Kibar E.A., Us F. (2014). Evaluation of structural properties of cellulose ether-corn starch based biodegradable films. Int. J. Polym. Mater..

[78] Zhang Y., Guo Q., Feng N., Wang J.-R., Wang S.-J., He Z.-H. (2016). Characterization of A- and B-type starch granules in Chinese wheat cultivars. J. Integr. Agric..

[79] Paranthaman R., Moses J.A., Anandharamakrishnan C. (2022). Powder X-ray diffraction conditions for screening curcumin in turmeric powder. J. Food Meas. Charact..

[80] Pandey K.U., Dalvi S.V. (2019). Understanding stability relationships among three curcumin polymorphs. Adv. Powder Technol..

[81] Montoya-Escobar N., Ospina-Acero D., Velásquez-Cock J.A., Gómez-Hoyos C., Serpa Guerra A., Gañan Rojo P.F., Vélez Acosta L.M., Escobar J.P., Correa-Hincapié N., Triana-Chávez O. (2022). Use of Fourier series in X-ray diffraction (XRD). Analysis and Fourier-Transform Infrared Spectroscopy (FTIR) for estimation of crystallinity in cellulose from different sources. Polymers.

[82] An D., Li H., Li D., Zhang D., Huang Y., Obadi M., Xu B. (2022). The relation between wheat starch properties and noodle springiness: From the view of microstructure quantitative analysis of gluten-based network. Food Chem..

[83] Jia R., Cui C., Gao L., Qin Y., Ji N., Dai l., Wang Y., Xiong L., Shi R., Sun Q. (2023). A review of starch swelling behavior: Its mechanism, determination methods, influencing factors, and influence on food quality. Carbohydr. Polym..

[84] Algar A.F.C., Umali A.B., Tayobong R.R.P. (2019). Physicochemical and functional properties of starch from Philippine edible Canna (*Canna indica* L.) rhizomes. J. Microbiol. Biotechnol. Food Sci..

